# Clinical and Pathological Data of 17 Non-Epithelial Pancreatic Tumors in Cats

**DOI:** 10.3390/vetsci7020055

**Published:** 2020-04-27

**Authors:** Katrin Törner, Marlies Staudacher, Katja Steiger, Heike Aupperle-Lellbach

**Affiliations:** 1 LABOKLIN GmbH & Co. KG, 97688 Bad Kissingen, Germany; aupperle@laboklin.de; 2 Tierärztliche Klinik Dr. Staudacher, 52078 Aachen, Germany; m.staudacher@tgz-aachen.de; 3 Institute of Pathology, Technische Universität München, 81675 Munich, Germany; katja.steiger@tum.de

**Keywords:** fPLI, DGGR lipase, histopathology, lymphoma, mast cell tumor, sarcoma

## Abstract

Tumors of mesenchymal origin are rarely reported in the pancreas. Therefore, this study characterized 17 feline non-epithelial pancreatic tumors, including clinical data, histopathology, and immunohistochemistry. Seventeen feline pancreatic tissue samples were investigated histopathologically and immunohistochemically. Selected pancreatic and inflammatory serum parameters, e.g., feline pancreatic lipase immunoreactivity (fPLI), 1,2-o-dilauryl-rac-glycero-3-glutaric acid-(6′-methylresorufin) ester (DGGR) lipase and serum amyloid A (SAA), were recorded, when available. The neoplasms were characterized as round (n = 13) or spindle (n = 4) cell tumors. Round cell tumors included 12 lymphomas and one mast cell tumor in ectopic splenic tissue within the pancreas. Lymphomas were of T-cell (n = 9) or B-cell (n = 3) origin. These cats showed leukocytosis (3/3) and increased fPLI (5/5), DGGR lipase (3/5) and SAA (4/5) values. Spindle cell tumors included two hemangiosarcomas, one pleomorphic sarcoma and one fibrosarcoma. The cat with pleomorphic sarcoma showed increased SAA value. Overall survival time was two weeks to seven months. These are the first descriptions of a pancreatic pleomorphic sarcoma and a mast cell tumor in accessory spleens within feline pancreas. Although rare, pancreatic tumors should be considered in cats presenting with clinical signs and clinical pathology changes of pancreatitis. Only histopathology can certainly distinguish solitary pancreatitis from a neoplasm with inflammation.

## 1. Introduction

Pancreatic neoplasms are rare in cats, dogs [1,2,3], and humans [4]. Carcinoma is the most common tumor type in cats and dogs, but adenomas, sarcomas, lymphomas, unclassified tumors, and metastatic neoplasms have also been described [1,2,3,5]. Pancreatic neoplasms generally cause non-specific constitutional and gastrointestinal signs such as lethargy, anorexia, weight loss, diarrhea, vomiting, abdominal pain, or palpable abdominal masses [6,7,8].

Lymphomas are common neoplasms in most domestic species. Gastrointestinal lymphoma is the most common form of lymphoma in cats and is associated with clinical presentation such as weight loss, vomiting, diarrhea anorexia, and lethargy [9]. However, pancreatic involvement has been reported in only some cases and pancreas-specific blood parameters were not included in the studies [10,11,12,13]. While retroviral diseases (feline leukemia virus (FeLV) and feline immunodeficiency virus) are risk factors, a high number of cats with alimentary lymphomas have been reported to be virus negative [14].

As a mutant of FeLV, the feline sarcomavirus can cause sarcomas in cats [15], but literature about feline pancreatic sarcomas is scant. There is one case report of a feline primary pancreatic carcinosarcoma with metastases in uterus, omentum, and diaphragm [16]. Mesenchymal pancreatic tumors in humans are reported to have a poor prognosis with a median overall survival time of 21 months [17,18]. Visceral hemangiosarcomas are a common neoplasm in dogs, especially German Shepherds, but are uncommon in cats [19]. Hemangiosarcomas in dogs often occur in the spleen and the right atrium [19]. In cats, they arise mostly in liver, intestine, lymph nodes, or spleen [20]. The prognosis is poor in both dogs and cats [19,20,21].

The aim of this study is to characterize 17 cases of feline non-epithelial pancreatic tumors including clinical data, histopathology, and immunohistochemistry.

## 2. Materials and Methods

During the 2011–2018 period, 571 feline pancreatic tissue samples, submitted routinely to LABOKLIN GmbH & Co. KG (Bad Kissingen, Germany), were investigated. From these, 540 showed representative sample size and adequate tissue preservation. Included into this study were mesenchymal tumors within the pancreas, like spindle and round cell tumors from 17 cats. Excluded were samples without (n = 31), other tumors such as primary exocrine neoplasms (21/540 adenomas, 54/540 carcinomas) or endocrine tumors (2/540), as well as tumor-like lesions like nodular hyperplasia.

The age of the cats was one to 17 years (median 12). Ten cats were male (1 entire, 9 neutered) and seven female (3 entire, 4 spayed). The breeds were Domestic Shorthair (DSH, n = 12), British Longhair (n = 1), British Shorthair (n = 1), Carthusian (n = 1), Siamese (n = 1), and Sacred Birman (n = 1).

Complete pancreas (n = 4), fully excised tumor masses (n = 4) and excisional biopsies (n = 9) of the pancreas were examined. The samples were measured and inspected macroscopically in detail with respect to size, cut surface, and color. This study refers to size of the complete neoplasm unless specified otherwise. Representative sites were prepared for routine histopathological examination and embedded in paraffin according to standard procedures. Slides were routinely stained with hematoxylin-eosin (HE) and Giemsa. Tumors were evaluated according to the world health organization (WHO) classification of mesenchymal [22,23,24] and hematopoietic tumors [25]. Mitotic figures were counted within 10 high power fields (HPF) by 400 x microscope objective (visual field: 68,700 µm²) in areas of the highest mitotic activity and were reported as mean value/HPF. Lymphomas were graded as low grade: <5 mitotic figures/HPF, intermediate grade: 5–10 mitotic figures/HPF and high grade: >10 mitotic figures/HPF [26]. Pancreatitis was classified according to De Cock et al. [27].

The immunohistochemical panel of primary antibodies used and their dilution are listed in Table 1. The two antibodies for FeLV (gp70 and p27) were diluted appropriately and consequently mixed at a ratio of 1:1 for further procedure. Pre-treatment included peroxidase blocking with 0.66% hydrogen peroxidase for all markers. Afterwards, the slides were heated either in 1:10 diluted HIER T-EDTA buffer pH 9.0 (10×) (Zytomed Systems GmbH, Berlin, Germany, 2VCO29-500) in a steam cooker (vimentin, desmin, CD31, CD3, CD79a, CD117/c-kit, FeLV), or in 1:10 diluted target retrieval solution pH 6.0 (10×) (Dako, Glostrup, Denmark, S1699) in a pressure cooker (smooth muscle actin, von Willebrand factor (vWF)). Dako EnVision+ System-HPR for mouse or rabbit primary antibodies (Dako, Glostrup, Denmark, K4006 or K4010) with 3,3‘-diaminobenzine (DAB) was used for immunohistochemical staining. Counterstaining was done with Meyer’s hematoxylin (Bio-Optica Milano S.p.A., Milano, Italy, BIO 05-06002/CO). Slides of the same organs prepared with normal mouse IgG (Dako, Glostrup, Denmark, X0931) or rabbit IgG (Dako, Glostrup, Denmark, X0936), with the same dilution as the specific reagents, were used as negative control. Positive controls varied for the different antibodies: tissue with fibrocytes and fibroblasts for vimentin, intestine for smooth muscle actin, muscle tissue for desmin, vessel rich tissue for vWF and CD31, lymph nodes for CD3 and CD79a, skin tissue with mast cells for CD117/c-kit, and PCR positive spleen tissue for FeLV.

Clinicopathologic data were available for six cats (five lymphomas and one sarcoma). The serum samples in these cases were taken shortly before or during surgery. Alpha-amylase (reference interval: <1850 U/L), 1,2-o-dilauryl-rac-glycero-3-glutaric acid-(6′-methylresorufin) ester (DGGR) lipase (reference interval: <26 U/L) and serum amyloid A (SAA, reference interval: <6.7 µg/mL) were examined with cobas e602 or cobas c701 analyzer (Roche Diagnostics, Mannheim, Germany). Feline pancreatic lipase immunoreactivity (fPLI, reference interval: <3.5 µg/L, questionable: 3.5–5.4 µg/L, indicative for pancreatitis: >5.4 µg/L) and feline trypsin-like immunoreactivity (fTLI, reference interval: 12–82 µg/L, indicative for pancreatitis >100 µg/L) values were determined by an ELISA assay at LABOKLIN GmbH & Co. KG.

## 3. Results

In pancreatic tissue from 17 cats, round (n = 13) and spindle (n = 4) cell tumors were diagnosed histopathologically (Table 2). Round cell tumors included 12 lymphomas and one mast cell tumor. Spindle cell tumors were classified as two hemangiosarcomas, one pleomorphic sarcoma, and one fibrosarcoma. Clinical pathology was available for five cats with lymphoma and one cat with pleomorphic sarcoma (Table 3).

### 3.1. Round Cell Tumors, n = 13

#### 3.1.1. Lymphoma, n = 12

Cats with lymphomas were one to 16 years old (median 11) and predominantly male (75.0%). The majority of cats were Domestic Shorthair (66.7%) (Table 2). Clinical reports were available for 9 out of 12 cats: The cats showed vomiting (n = 3), lethargy (n = 3), inappetence (n = 3), diarrhea (n = 2), painful abdomen (n = 2), ascites (n = 2), palpable abdominal mass (n = 1), and seizures (n = 1). Clinical pathology results were available for five cats (Table 3). The fPLI values were increased in all cats (5/5) and SAA values were high in four out of five cats. DGGR lipase values were increased in three out of five cases. Alpha-amylase and fTLI were within the reference range in all cats (5/5). A complete blood count (CBC) was available for three cats: leukocytosis was present in all animals, but lymphocytosis with severe leukocytosis and neutrophilia only in one cat (No. 7). One 10-year-old British Shorthair (No. 3) showed lymphopenia.

**Figure 1:**
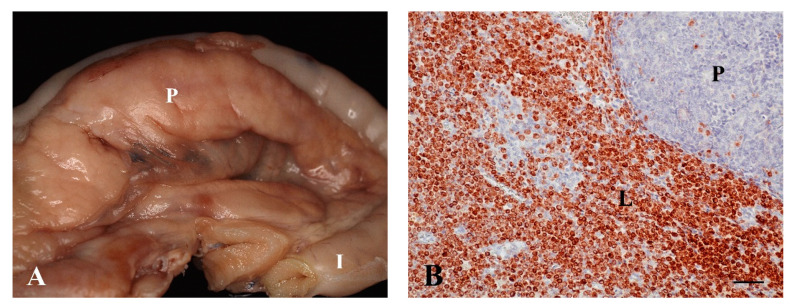
(**A**) T-cell lymphoma. The pancreas (P) close to the small intestine (I) is enlarged, white and firm. (16-year-old, male neutered Domestic Shorthair, No. 7, formalin fixed tissue); (**B**) T-cell lymphoma with intense CD3-expression of neoplastic lymphocytes (L) in the pancreas (P). (15-year-old, male neutered Domestic Shorthair, No. 9, IHC, CD3. Bar, 50 µm).

The macroscopic appearance of pancreatic tissues varied from normal to diffusely enlarged with fine granulated texture (Figure 1A). Masses varied in size from 0.3 × 0.3 × 0.3 to 4.0 × 4.0 × 4.0 cm. Histopathologically, ten cats with lymphoma showed additional involvement of the intestine (n = 6), lymph node (mesenteric, n = 3; unknown location, n = 1), adipose tissue (mesenteric, n = 2; peripancreatic, n = 1), liver (n = 2), omentum (n = 2), stomach (n = 1), spleen (n = 1) or kidney (n = 1). Histologically, neoplastic lymphocytes infiltrated the pancreas multifocally. Neoplastic cells were small (n = 1), intermediate (n = 4), or large (n = 7) sized. In contrast to lymphoplasmacytic pancreatitis, the tumor cell population was monomorphic, with varying degrees of nuclear atypia and mitotic activity. Lymphomas were graded as low (n = 9), intermediate (n = 2), or high (n = 1) grade. In seven out of 12 cases, an additional mild purulent (n = 3), mild lymphoplasmacytic (n = 2) or marked purulent (n = 2) pancreatitis was present.

Lymphoma was diagnosed in most of the cases (11/12) with the standard staining (HE). However, case No. 12 showed undifferentiated malignant neoplasia and immunohistochemistry was necessary for the diagnosis. Immunohistochemically, lymphomas were classified as T-cell (n = 9, Figure 1B) or B-cell (n = 3) lymphoma. In two cats with T-cell lymphoma (Nos. 5, 11), FeLV was detected. The remaining ten cats were tested negative.

Seven cats were euthanized during surgery. A one-year-old Domestic Shorthair (No. 1) had a survival time of two months and a 13-year-old Siamese (No. 4) survived seven months. Treatment data for these cases were not available.

#### 3.1.2. Mast Cell Tumor, n = 1

**Figure 2:**
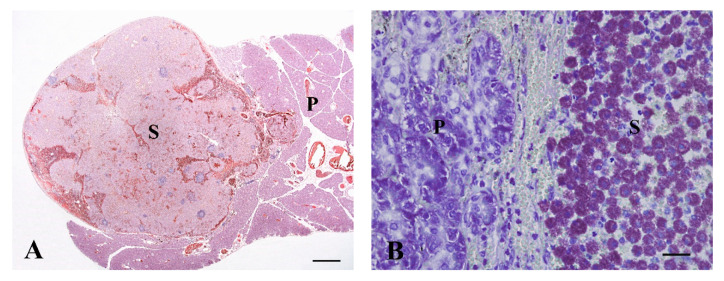
(**A**) Mast cell tumor. The accessory spleen is prominent as a mass (S) within the pancreas (P). (16-year-old, female spayed British Longhair, No. 13, HE. Bar, 1000 µm); (**B**) The accessory spleen (S) within the pancreas (P) is diffusely infiltrated by lots of mast cells (the same cat as in (**A**), Giemsa stain. Bar, 25 µm).

A 16-year-old female British Longhair cat (No. 13) was presented to the clinician for routine vaccination, and during palpation a mass in the abdomen was identified. Ultrasonography showed marked splenomegaly. During laparotomy, a diffusely enlarged (22.0 × 7.5 × 4.0 cm) spleen was removed. Two dark brown masses (0.3 × 0.3 × 0.3 and 1.0 × 0.6 × 0.5 cm) were found within the pancreas. Microscopically they were identified as accessory spleens. Both, spleen and accessory spleens showed marked diffuse infiltration by well-differentiated large mast cells (Figure 2A). Nuclear atypia and anisokaryosis were mild. The mitotic count was 1/HPF. No vessel infiltration could be observed, but some mast cells infiltrated the mildly hyperplastic pancreatic tissue. Giemsa staining showed intense metachromatic granularity of mast cells (Figure 2B). However, there was no c-kit expression detectable. FeLV testing was negative. No further treatment was performed. The cat was euthanized 2 months after diagnosis due to progressive renal and cardiac insufficiency.

### 3.2. Spindle Cell Tumors, n = 4

**Figure 3:**
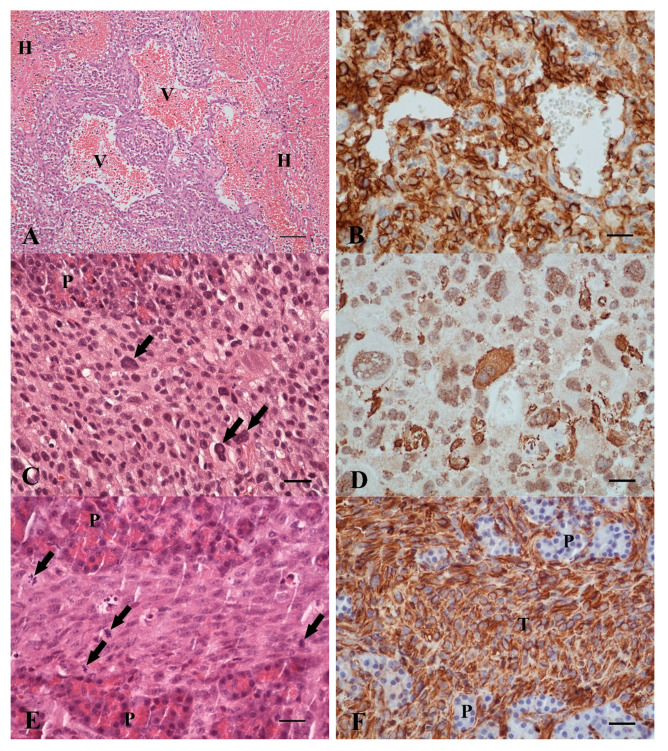
(**A**) Hemangiosarcoma. Tumor cells form vascular spaces (V) and there is marked inflammation and hemorrhage (H) (12-year-old, female spayed Domestic Shorthair, No. 15. HE. Bar, 100 µm); (**B**) hemangiosarcoma. Intense CD31 expression in neoplastic cells. (same case as (**A**), IHC, CD31. Bar, 25 µm); (**C**) pleomorphic sarcoma. Pleomorphic, poorly differentiated spindle cells with single giant nuclei (arrow) in the pancreas (P) (17-year-old, female Domestic Shorthair, No. 16. HE. Bar, 25 µm); (**D**) pleomorphic sarcoma. Varying expression intensity of smooth muscle actin in pleomorphic tumor cells. (Same case as (**C**) (IHC, actin. Bar, 25 µm); (**E**) fibrosarcoma. Mitotic figures (arrows) and intense anisokaryosis of neoplastic spindle cells between exocrine pancreatic acini (P). (13-year-old, male neutered Domestic Shorthair, No. 17, HE. Bar, 25 µm); (**F**) fibrosarcoma. Intense vimentin expression by the tumor cells (T) infiltrating the pancreas (P) without vimentin expression (same case as (**E**), IHC, vimentin. Bar, 25 µm).

In two cats (Nos. 14, 15), pancreatic masses were hemangiosarcomas (Table 2). One cat was a 13-year-old Domestic Shorthair (No. 14) with a palpable abdominal mass. Clinical findings in Cat No. 15 were not reported. Blood samples were not investigated in these cases. In both cats, histopathology showed infiltrative vasoformative growth of neoplastic endothelial cells, marked hemorrhage, thrombosis, mixed inflammation, and necrosis in the pancreas (Figure 3A). Mitotic count was 1/HPF and 3/HPF, respectively. The neoplastic cells co-expressed vimentin, vWF and CD31 (Figure 3B). They were tested negative for FeLV. Both cats were euthanized during surgery.

One 17-year-old Domestic Shorthair (No. 16) was lethargic and had a palpable abdominal mass. Pancreas-specific results were within reference intervals, but the SAA value was increased and CBC revealed mild leukocytosis with neutrophilia and lymphopenia (Table 3). Multiple non-encapsulated firm masses (0.1 × 0.1 × 0.1 to 2.0 × 2.0 × 2.0 cm) within the pancreas were noted. Further neoplastic masses were not identified by clinical investigation of the skin or during laparotomy. Microscopically, the multinodular pleomorphic sarcoma with myofibroblastic differentiation was characterized by poorly differentiated spindle cells as well as pleomorphic and multinucleated giant cells within the pancreatic interstitium (Figure 3C). Mitotic count was 9/HPF. Vascular infiltration was present. There were multifocal mild mixed inflammation and small areas of necrosis. The pleomorphic sarcoma showed a diffuse expression of vimentin and inhomogeneous co-expression of actin (Figure 3D). Other sarcomas such as rhabdomyosarcoma, gastrointestinal stromal tumor, or mast cell tumor were excluded by negative immunohistochemical reaction with anti-desmin and c-kit antibodies and Giemsa stain negative. Furthermore, FeLV immunohistochemistry was negative. The cat was euthanized during surgery.

One 13-year-old Domestic Shorthair (No. 17) was presented with poor appetite and ascites. No other intraabdominal masses or cutaneous sarcomas were noted by the clinician. Macroscopically, the submitted tissue samples were brown and firm. Histopathologically, a fibrosarcoma with interstitial infiltrative growth of moderately differentiated neoplastic spindle cells with heterogeneous morphology (Figure 3E) was diagnosed. Mitotic count was 2 mitoses/HPF. Neoplastic cells exclusively expressed vimentin (Figure 3F). FeLV was negative. The cat was euthanized two weeks after surgery.

## 4. Discussion

Pancreatic neoplasms are rare in both humans [4] and domestic animals [3,28]. Epithelial solid and cystic pancreatic neoplasms have been previously described in a larger series of 70 cats [29]. Non-epithelial neoplasms (e.g., lymphoma, sarcoma) are even rarer in all species [1,3,30].

In general, lymphoma is the most common hematopoietic feline tumor [31]. A clinical diagnosis of gastrointestinal lymphoma can be challenging because of its unspecific clinical presentation, especially if neither masses nor lymphocytosis are present. During the clinical work up of such uncertain cases, clinical pathology is commonly performed. In our study, cats with lymphoma for which blood values were available (n = 5) showed high (n = 3) or questionable (n = 2) fPLI values, as well as increased DGGR lipase (n = 3) and SAA values (n = 4). This may be explained by an accompanying purulent pancreatitis in four cases. Further studies about pancreas-specific laboratory parameters in cats with lymphomas are necessary to investigate the prevalence of pancreatic involvement. Furthermore, inflammation was also reported in numerous feline epithelial pancreatic tumors [29] and as far as serological data were available, fPLI value was increased in several cases [32]. One may conclude that increased serological pancreatic enzymes can be indicative of an acute pancreatitis. However, varying underlying pancreatic neoplasms should be taken into consideration especially if empiric treatment is unsuccessful or if there is imaging evidence of neoplasia. Without histopathological investigation, pancreatic neoplasms may be misdiagnosed as an unsuccessfully treated pancreatitis. However, one cat with T-cell lymphoma (No. 7) showed increased fPLI and DGGR lipase values and normal SAA value without histological signs of pancreatitis. This is consistent with the literature, where false negative (sensitivity) and false positive (specificity) results have been described previously for serological pancreatitis diagnostic in some cases [33].

Morphological differentiation between inflammatory and neoplastic small lymphocytes in the pancreas may require further investigation. Immunohistochemical assessment is an important diagnostic tool [34]. In contrast, polymerase chain reaction for antigen receptor gene rearrangement analysis (PARR) is well-known to have some limitations in cats [35]. In our study, T-cell lymphomas were more frequent (75%) than B-cell lymphomas. This correlates with the adjoining anatomy of small intestine and pancreas as interstitial T-cell lymphomas are more frequent than B-cell lymphomas in cats [36]. Thus, the pancreatic sites of lymphomas were interpreted as part of a multicentric systemic disease. Immunohistochemistry for FeLV was positive for two cats with T-cell lymphoma (No. 5, 11). This is in accordance with the literature, where retroviral diseases (FeLV or feline immunodeficiency virus) were listed as a risk factor in lymphomas [14]. In conclusion, without cytological/histopathological examination and/or imaging techniques, feline pancreatic lymphoma is easily missed if solitary pancreatitis is suspected because of clinical pathology and non-specific clinical findings. This has a strong potential impact on therapy and prognosis, especially considering the treatability of lymphomas by chemotherapy. Survival time of lymphoma subtypes may vary. Sato et al. [37] showed a shorter survival time in alimentary (48 days) lymphomas than in cats with nasal (135 days) or mediastinal (143 days) lymphomas. Wolfesberger et al. [38] reported the highest survival time in intestinal T-cell lymphomas (1.7 years) compared to large B-cell lymphoma (4.5 months) or peripheral T-cell lymphoma (6.1 months). In our study, seven out of 12 cats with lymphomas were either euthanized or died during or shortly after surgery. Only two cats with lymphomas lived on for 2 months (No. 1, B-cell lymphoma) and 7 months (No. 4, T-cell lymphoma), respectively.

Feline mast cell tumors are predominantly cutaneous neoplasms but can also appear in the spleen [24]. In our study, a mast cell tumor in accessory splenic tissue was described for the first time. Accessory splenic tissue is usually an incidental finding during laparotomy and appears as small dark brown masses within the pancreas [39]. Ectopic splenic tissue was seen in 24 out of 540 cases (4%) of routinely submitted feline pancreas samples in our laboratory (unpublished data). As in our case (cat No. 13), a high percentage of mast cell tumors in cats are negative for different immunohistochemical markers, including c-kit [40], which can impede a diagnosis in poorly differentiated cases. Prognostic factors are not entirely clear in cats, but splenectomy has been recommended for feline splenic mast cell tumors [41,42]. Careful exploration of the pancreas is recommended because of the risk of incomplete resection of splenic tissue in the case of affected accessory spleens, as in our case.

Sarcomas of the feline pancreas are very rare. Because of the retrospective nature of our study, there were incomplete data on signalment, therapy, and clinical outcome. Previous clinical and surgical reports were often unavailable. Therefore, a distinction between those sarcomas that were primary pancreatic neoplasms and metastases from elsewhere cannot be made with certainty in all cases. Nevertheless, because of the tumors being exclusively reported within the pancreas, both macroscopically and histologically, there is a high likelihood of them being primaries.

Feline visceral hemangiosarcomas were mostly seen in the liver, intestine, and lymph nodes but rarely (2/26 cats) in the pancreas [20]. Both cats with hemangiosarcomas included in our study died or were euthanized shortly after surgery. According to the literature, prognosis for feline visceral hemangiosarcoma with a multifocal presentation is poor, with a median survival time of 77 days [20]. Correlation between the degree of differentiation, mitotic rate or size of feline hemangiosarcoma and the clinical outcome could not be found [43].

The present study included a fibrosarcoma and a pleomorphic sarcoma in the feline pancreas. Nomenclature of pleomorphic sarcomas is still controversial. The terms “undifferentiated pleomorphic sarcoma,” “anaplastic sarcoma with giant cells,” or “malignant fibrous histiocytoma” are used synonymously in veterinary pathology [15]. In cat No. 16, immunohistochemistry revealed small areas of myofibroblastic differentiation within the neoplasm. In literature, this combination of histological type of sarcoma and immunohistochemical expression pattern has been described for feline cutaneous post-vaccinal sarcomas [44,45]. Cutaneous sarcomas may undergo malignant transformation of fibrocytes due to an inflammation [46]. Furthermore, there is one case report of a fibrosarcoma arising at the site of a retained surgical sponge in a cat [47]. In human medicine, inflammatory pseudotumors have been reported as a benign change in various organs, predominantly in the lungs [48]. The term “inflammatory fibrosarcoma” was suggested for some of these cases with metastases [49]. The cat with the pleomorphic sarcoma in our study had increased SAA values of an unknown cause and the pathogenesis of the tumor remained unclear. In human medicine, recurrence of metastatic sarcomas in the pancreas is common and a median overall survival time of 21 months [17] as well as moderate complication rates [18] have been described. The cats in our study were euthanized during surgery because of the poor prognosis.

## 5. Conclusions

In summary, our study described the characteristics of five different pancreatic tumor types of round or spindle cell origin in 17 cats. It includes the first descriptions of a pancreatic pleomorphic sarcoma and a mast cell tumor in accessory spleens within the pancreas. Epithelial pancreatic neoplasms have been reported previously [29] and metastases of other carcinomas may affect the pancreas also (unpublished data). In general, pancreatic neoplasms may be accompanied by inflammation, and histopathological investigation is required to differentiate pancreatitis from an inflamed pancreatic neoplasm. This may have a strong impact on therapy and prognosis.

## Figures and Tables

**Table 1:** Primary antibodies and pretreatment used in immunohistochemistry of 17 feline non-epithelial pancreatic tumors.

Marker	Type of Antibody	Source	Dilution	Pretreatment
Vimentin	Monoclonal mouse anti-vimentin (Clone V9)	Dako, Glostrup, Denmark, M0725	1:1000	Peroxidase blocking; steam cook in EDTA buffer
Smooth muscle actin	Monoclonal mouse anti-human (Clone 1A4)	Dako, Glostrup, Denmark, M0851	1:100	Peroxidase blocking; pressure cook in citrate buffer
Desmin	Monoclonal mouse anti-human (Clone D33)	Dako, Glostrup, Denmark, M0760	1:100	Peroxidase blocking; steam cook in EDTA buffer
Von Willebrand factor	Polyclonal rabbit anti-human	Dako, Glostrup, Denmark, A0082	1:2000	Peroxidase blocking; pressure cook in citrate buffer
CD31	Monoclonal mouse anti-human (Clone JC70A)	Dako, Glostrup, Denmark, M0823	1:100	Peroxidase blocking; steam cook in EDTA buffer
CD3	Monoclonal mouse anti-human (Clone F7.2.38)	Dako, Glostrup, Denmark, M7254	1:100	Peroxidase blocking; steam cook in EDTA buffer
CD79a	Monoclonal mouse anti-human (Clone HM57)	Bio-Rad Laboratories Inc, Munich, Germany, MCA2538GA	1:3000	Peroxidase blocking; steam cook in EDTA buffer
CD117/c-kit	Polyclonal rabbit anti-human	Dako, Glostrup, Denmark, A4502	1:150	Peroxidase blocking; steam cook in EDTA buffer
Feline Leukemia Virus gp70	Monoclonal mouse anti-feline leukemia virus (Clone C11D8)	Bio-Rad Laboratories Inc, Munich, Germany, MCA1897	1:200	Peroxidase blocking; steam cook in EDTA buffer
Feline Leukemia Virus p27	Monoclonal mouse anti-feline leukemia virus (Clone PF12J-10A)	Bio-Rad Laboratories Inc, Munich, Germany, MCA2551	1:100	Peroxidase blocking; steam cook in EDTA buffer

**Table 2:** Signalment, histopathological/immunohistochemical data and clinical outcome of cats with non-epithelial tumors (n = 17).

Case No.	Age (years)	Sex	Breed	Clinical Signs	Pancreatic Sample Size (cm)	Diagnosis	Mi/ HPF	Further Infiltrated Organs	Staining, IHC Expression		Survival Time
									+	−	
1	1	MN	DSH	Inappetence, ascites	TS 0.8 × 0.3 × 0.3	Large BCL	13	Kidney, liver, LN	CD79a	CD3, FeLV	2 months
2	12	F	SB	Inappetence	CM 3.0 × 3.0 × 3.0	Large BCL	5	Om	CD79a	CD3, FeLV	U
3 *	10	MN	BSH	Vomiting, inappetence, palpable abd. mass	CM 4.0 × 4.0 × 4.0	Large BCL	9	Stomach, mAT	CD79a	CD3, FeLV	Eutha
4	13	MN	Si	Vomiting, diarrhea	TS 1.0 × 0.4 × 0.3	Small TCL	0	Intestine	CD3	CD79a, FeLV	7 months
5 *	15	MN	DSH	Lethargy, painful abd.	TS 1.5 × 1.5 × 1.5	Intermediate TCL	0	Intestine, mLN	CD3, FeLV	CD79a	Eutha
6	11	F	DSH	Diarrhea	TS 0.3 × 0.3 × 0.3	Intermediate TCL	0	-	CD3	CD79a	Eutha
7 *	16	MN	DSH	Lethargy, ascites	CP 8.5 × 7.0 × 2.6	Intermediate TCL	3	Intestine, pAT, mLN	CD3	CD79a, FeLV	Eutha
8	10	MN	DSH	U	CP 9.0 × 6.0 × 3.0	Intermediate TCL	4	Liver, intestine, mLN	CD3	CD79a	Eutha
9 *	15	MN	DSH	Lethargy, seizures	CP 7.0 × 6.5 × 0.5 with two masses 0.5 × 0.5 × 0.5	Large TCL	1	Intestine	CD3	CD79a, FeLV	Eutha
10	11	M	Cart	U	Two TS 1.0 × 0.5 × 0.1 and 1.1 × 0.5 × 0.4	Large TCL	3	Om, spleen	CD3	CD79a, FeLV	Eutha
11	8	FS	DSH	U	Two TS 3.4 × 3.0 × 3.0 and 5.0 × 3.4 × 2.4	Large TCL	3	Intestine, mAT	CD3, FeLV	CD79a	U
12 *	10	MN	DSH	Vomiting, painful abd.	TS 0.4 × 0.4 × 0.4	Large TCL	4	-	CD3	CD79a, FeLV	U
13	16	F	BLH	Palpable abd. mass	CM 0.3 × 0.3 × 0.3 and 1.0 × 0.6 × 0.5	Mast cell tumor	1	Spleen	Giemsa	c-kit, FeLV	2 months
14	13	FS	DSH	Palpable abd. mass	CM 2.0 × 1.3 × 1.0	Hemangio-sarcoma	3	-	Vim, vWF, CD31		Eutha
15	12	FS	DSH	U	TS 5.5 × 2.0 × 0.4	Hemangio-sarcoma	1	Om, intestine, LN	Vim, CD31, vWF		Eutha
16 *	17	F	DSH	Palpable abd. mass, lethargy	CP 17.0 × 9.0 × 5.0 with multiple masses up to 2.0 × 2.0 × 2.0	PS with myofibro-blastic parts	9	Om, adherent on capsule of spleen, intestine, liver	Vim, actin	desmin, c-kit	Eutha
17	13	MN	DSH	Inappetence, ascites	Two TS 1.2 × 0.8 × 0.6 and 3.0 × 3.0 × 2.0	Fibrosarcoma	2	Spleen	Vim		2 weeks

− = no/negative, + = positive, * = clinical pathology is available, abd. = abdominal/abdomen, BCL = B-cell lymphoma, BLH = British Longhair, BSH = British Shorthair, Cart = Carthusian, CM = complete mass, CP = complete pancreas, DSH = Domestic Short Hair, Eutha = euthanasia after surgery, F = female, FeLV = feline leukemia virus, F = female spayed, HPF = high power field, IHC = immunohistochemistry, LN = lymph node, M = male, Mi = mitoses, mAT = mesenteric adipose tissue, mLN = mesenteric lymph node, MN = male neutered, Om = omentum, pAT = peripancreatic adipose tissue, PS = pleomorphic sarcoma, SB = Sacred Birman, Si = Siamese, TCL = T-cell lymphoma, TS = tissue sample, U = unknown, Vim = vimentin, vWF = von Willebrand factor.

**Table 3:** Clinical pathology data of cats with non-epithelial pancreatic tumors (n = 6).

Case No.	Diagnosis	fPLI (<3.5 µg/ L)	fTLI (12–82 µg/ L)	DGGR Lipase (<26 U/L)	Alpha Amylase (<1850 U/L)	SAA (<6.7 µg/mL)	Leuko (6.0–11.0 G/L)	Neutro (3.0–11.0 G/L)	Lymph (1.0–4.0 G/L)
3	BCL, mild purulent pancreatitis	4.7	30.7	25.9	922	128.83	16.1	15.0	0.6
5	TCL, mild purulent pancreatitis	4.2	25.6	30.1	552	41.06			
9	TCL, mild purulent pancreatitis	5.6	33.0	15.4	891	47.51	11.4	9.2	1.0
12	TCL, mild purulent pancreatitis	22.9	43.2	40.6	839	62.40			
7	TCL, mild lymphocytic pancreatitis	>40.0	14.1	268.7	405	4.78	53.1	31.9	10.1
16	Pleomorphic sarcoma, mild mixed pancreatitis	1.8	38.5	8.7	701	86.12	19.3	18.5	0.4

BCL = B-cell lymphoma, DGGR = 1,2-o-dilauryl-rac-glycero-3-glutaric acid-(6′-methylresorufin) ester, fPLI = feline pancreatic lipase immunoreactivity, fTLI = feline trypsin-like immunoreactivity, Leuko = leukocytes, Lymph = lymphocytes, Neutro = neutrophilic granulocytes, SAA = serum amyloid A, TCL = T-cell lymphoma.
